# Circulating Regulatory T-Cells in Monoclonal Gammopathies of Uncertain Significance and Multiple Myeloma: In Search of a Role

**DOI:** 10.1155/2016/9271469

**Published:** 2016-07-14

**Authors:** Giovanni D'Arena, Giovanni Rossi, Luca Laurenti, Teodora Statuto, Fiorella D'Auria, Luciana Valvano, Vittorio Simeon, Aldo Giudice, Idanna Innocenti, Vincenzo De Feo, Rosanna Filosa, Pellegrino Musto

**Affiliations:** ^1^Hematology and Stem Cell Transplantation Unit, IRCCS Cancer Referral Center of Basilicata, 85028 Rionero in Vulture, Italy; ^2^Hematology Unit, IRCCS “Casa Sollievo della Sofferenza” Hospital, 71013 San Giovanni Rotondo, Italy; ^3^Hematology Chair, Catholic University of “Sacred Heart”, 00168 Rome, Italy; ^4^Laboratory of Clinical Research and Advanced Diagnostics, IRCCS Cancer Referral Center of Basilicata, 85028 Rionero in Vulture, Italy; ^5^Laboratory of Preclinical and Translational Research, IRCCS Cancer Referral Center of Basilicata, 85028 Rionero in Vulture, Italy; ^6^Epidemiology Unit, National Cancer Institute of Naples “G. Pascale Foundation”, IRCCS, 80131 Naples, Italy; ^7^Pharmacology Department, University of Salerno, 84084 Salerno, Italy; ^8^Department of Experimental Medicine, Second University of Naples, 80138 Naples, Italy; ^9^Scientific Direction, IRCCS Cancer Referral Center of Basilicata, 85028 Rionero in Vulture, Italy

## Abstract

The frequency and function of regulatory T-cells (Tregs) in multiple myeloma (MM) are still matter of debate. The percentage and absolute number of circulating Tregs (CD4^+^CD25^+high  density^CD127^−/low  density^) from 39 patients with untreated MM and 44 patients with monoclonal gammopathies of uncertain significance (MGUS) were tested and compared with 20 healthy subjects as controls. The mean percentage number of circulating Tregs was 2.1%  ± 1.0 (range 0.75–6.1%) in MM patients; 2.1%  ± 0.9 (range 0.3–4.4%) in MGUS; and 1.5%  ± 0.4 (range 0.9–2.1%) in controls (*p* ns). Mean absolute number of Tregs was 36.3/*μ*L ± 23.7 (range 6.7–149/*μ*L) in MM; 38.8/*μ*L ± 19.1 (range 4.3–87/*μ*L) in MGUS; and 39.4/*μ*L ± 12.5 (range 18–63/*μ*L) in controls (*p* ns). After a median follow-up of 38 months, 5 MGUS and 2 smoldering MM (SMM) transformed into overt MM; however Tregs number did not predict this evolution. With respect to MM patients and after a median follow-up of 33 months, Tregs did not show any significant correlation with main clinical and laboratory characteristics. Finally, from a functional point of view, Tregs displayed an effective suppressor function, irrespective of disease status. This study indicates that the number of circulating Tregs does not differ in different monoclonal gammopathies and normal subjects and do not correlate with clinical features of MM.

## 1. Introduction

Natural and inducible regulatory T-cells (Tregs) are a small subset of T-lymphocytes able to suppress immune responses by direct interaction with other immune cell types or through immunosuppressive cytokines [1]. They appear to be crucial in maintaining immune homeostasis, mediating peripheral tolerance and preventing autoimmunity. Emerging evidences suggest that Tregs may also modulate host T-cell activity against tumor-associated antigens, thereby facilitating tumor escape from immunological control [2].

Tregs are characterized by the constitutive expression of surface CD4 antigen, high levels of surface CD25 antigen (IL-2 receptor *α*-chain), and the transcription factor forkhead box P3 (FOXP3), with low to nil CD127 (IL-7 receptor) [3].

We have recently demonstrated that Tregs may have a prognostic role in patients with Rai stage 0 chronic lymphocytic leukemia [4]. However, frequency and function of Tregs in monoclonal gammopathies of uncertain significance (MGUS) and multiple myeloma (MM) are still matter of debate. Aiming to evaluate a possible prognostic significance in MGUS and MM, we studied the percentage and absolute number of circulating Tregs in a consecutive, prospective cohort of these patients and in a group of healthy subjects, as controls.

## 2. Materials and Methods

Methylenediaminetetraacetic acid- (EDTA-) anticoagulated peripheral blood samples from 39 patients with newly diagnosed MM (mean age 69 years; range 37–90 years; 18 male; 21 female) and 44 patients with MGUS (mean age 65 years; range 39–87 years; 25 male; 19 female) were collected after informed written consent. Ten out of 39 MM patients were reclassified as having smoldering MM (SMM) just after the diagnosis. No relapsed, treated, or in remission phase after therapy patients were evaluated. Peripheral blood samples from 20 healthy subjects (mean age 59 years; range 32–80 years; 10 male; 10 female) were used as controls. Main patients' characteristics are summarized in Table 1.

**Figure 1:**
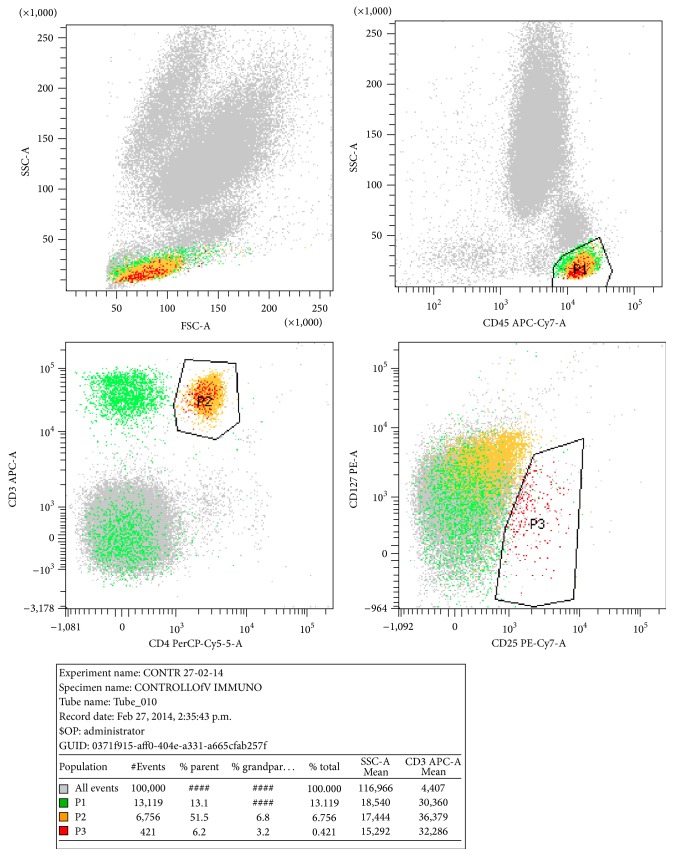
Flow cytometric analytical gating strategy. Tregs were evaluated, after selecting on gated CD45^+^ lymphocytes (P1), CD4^+^ cells (P2) as cells with CD25^+^ at high density and CD127 low density or undetectable levels (P3).

Tregs were numbered by means of flow cytometry (FACSCanto II, Becton Dickinson Biosciences (BDB), San Jose, CA, USA) as follows: 100 *μ*L of each of the following directly conjugated monoclonal antibodies (MoAbs): CD4-PerCP (peridinin chlorophyll protein complex)/CD127-PE (phycoerythrin)/CD25-Pe-Cy7 (PE-Cyanine 7)/CD45-APC-Cy/(allophycocyanin-Cy7). All MoAbs were purchased from BDB. After lysis of red blood cells and repeated washing, the samples were analyzed by flow cytometry, by acquiring a minimum of 20,000 events for each sample. All subsequent analyses were performed using FACS-Diva software (BDB). Tregs were evaluated according to the gating strategy protocol previously described (Figure 1) [12] and defined by the expression of CD4 and CD25 at high density and CD127 at low density or undetectable levels. This gating strategy was chosen because it better discriminates between Tregs and activated T-cells that normally upregulate CD25 antigen [3].

An* in vitro* proliferation inhibition assay, as previously described by Castella et al. [13], was used in 14 representative subjects (3 healthy controls and 4 MGUS, 3 SMM, and 4 active MM patients) to assess the suppressive function of circulating Tregs. Briefly, cell proliferation was evaluated measuring the uptake of 1 *μ*Ci ^3^HTdR, with a scintillation counter, by cell culture in which allogeneic PBMC were used as accessory cells, purified CD4^+^CD25^+^CD127^low^ cells as Tregs, autologous CD4^+^CD25^−^ cells as responder cells, and soluble anti-CD3 as polyclonal activator. Conventional CD4^+^CD25^−^ T-cells (T-conv): Treg ratio was 1 : 1. The percentage of inhibition was calculated as follows: 1 − [(average cpm counts in T responders + Tregs wells)]/[(average cpm counts in T responders wells)] × 100.

### 2.1. Statistical Analysis

Continuous variables are reported as means, standard deviations, and range. Comparisons were performed by use of single *t*-test or ANOVA depending on the number of group compared. The degree of association between two variables was measured using Spearman rank correlation for nonparametric data to assure the pertinence of all variables. Progression-free survival (PFS) was measured from time of diagnosis to time of progression into overt MM for MGUS and SMM or after an effective first-line therapy in MM patients; overall survival (OS) was determined from time of diagnosis to death event. The Kaplan-Meier method was used to analyze both PFS and OS. The receiver-operator-curve (ROC) analysis was performed to estimate cut-off of Tregs (as number or percentage) to predict progression or survival. Statistical significance was defined as *p* < 0.05. Statistical analyses were performed using R statistical software (version 3.2.0; R Foundation for Statistical Computing).

## 3. Results

**Figure 2:**
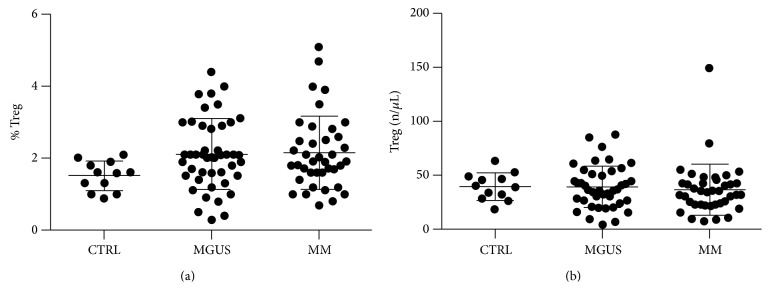
Circulating Tregs, detected as percentage of all lymphocytes (a) and as absolute number (b) in controls (CTRL), monoclonal gammopathy of uncertain significance (MGUS), and multiple myeloma (MM) patients, including those with smoldering MM (SMM). Horizontal bars indicate mean (± standard deviation) values. No statistically significant differences were found among the three groups.

Overall, the mean number of circulating Tregs, detected as percentage of all lymphocytes, was 2.1%  ± 1.0 (range 0.75–6.1%) in MM patients; 2.1%  ± 0.9 (range 0.3–4.4%) in MGUS; and 1.5%  ± 0.4 (range 0.9–2.1%) in controls (*p* ns) (Figure 2). Mean absolute number of Tregs was 36.3/*μ*L ± 23.7 (range 6.7–149/*μ*L) in MM; 38.8/*μ*L ± 19.1 (range 4.3–87/*μ*L) in MGUS; and 39.4/*μ*L ± 12.5 (range 18–63/*μ*L) in controls (*p* ns) (Figure 2). Patients with SMM were included within the MM group because of their mean percentage and absolute number levels for Tregs did not significantly differ from those of MGUS and active MM patients (data not shown).

After a median follow-up of 38 months (range 28–42 months) 5 MGUS and 2 SMM transformed into overt MM; however both percentage and absolute number of Tregs did not predict this evolution. Moreover, with respect to MM patients and after a median follow-up of 33 months (range 22–40 months), Tregs did not show any significant correlation with main clinical and laboratory characteristics, in particular with the type and amount of M-component, international staging system (ISS), bone marrow plasma cell infiltration, PFS, and OS (data not shown). As cytogenetic data were available only in a minority of MM patients, it was not possible to include this parameter in the analysis.

**Figure 3:**
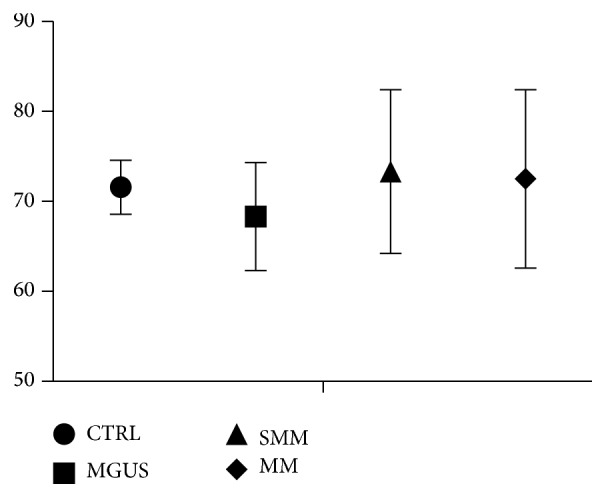
Percentage of inhibition of autologous CD4^+^CD25^−^ cells in healthy controls (CTRL) (71.6%  ± 3%), monoclonal gammopathy of uncertain significance (MGUS) (68.3%  ± 6%), smoldering multiple myeloma (SMM) (73.3%  ± 9.1%), and active multiple myeloma (MM) (72.5%  ± 9.9%) (*p* ns).

As shown in Figure 3, from a functional point of view, Tregs demonstrated to be able to inhibit autologous CD4^+^CD25^−^ cells at a mean rate of 71.6%  ± 3% in healthy controls, 68.3%  ± 6% in MGUS, 73.3%  ± 9.1% in SMM, and 72.5%  ± 9.9% in active MM (*p* ns).

## 4. Discussion

Though it has been hypothesized that Tregs accumulate in the course of MM exerting an immunosuppressive function, thus favoring the progression of the disease, conflicting results have been published so far on the frequency and prognostic relevance of Tregs in monoclonal gammopathies (Table 2) [5–11]. The variable results reported by different groups which have investigated Tregs in MM are at least in part probably due to the heterogeneous experimental approaches used: bone marrow, whole blood, isolated peripheral blood mononuclear cells (PBMC), depletion of CD25^+^ T-cells, different panel of monoclonal antibodies, and different analytical strategy to identify Tregs by means of flow cytometry (Table 2). In this setting, we choose to use CD127 evaluation, instead of FoxP3, to avoid technical bias due to permeabilization techniques and the subjectivity related to the variable expression of CD25 [3]. Furthermore, in our study, we analyzed peripheral blood. This must be taken into account because bone marrow Tregs frequency may differ from that found in peripheral blood. In fact, it has been reported that MM microenvironment support overexpression of FOXP3 and CTLA4 expression in bone marrow, thus suggesting an accumulation of immunosuppressive Tregs in the tumor microenvironment of MM patients [14]. The tumor growth and the failure of local immune control may be favored by changes in the bone marrow microenvironment, such as the accumulation of Tregs that may contribute to the immune imbalance in MM. However, in some studies, frequencies and total counts of Tregs were found similar in the bone marrow and peripheral blood of MM patients and control subjects [11].

In almost all functional studies, Tregs activity was found comparable to that of healthy subjects, with the exception of the Prabhala et al. study [5]. In a critical revision published by Muthu Raja et al., this was attributed to the use of PBMC as responder cells [15]. From a functional point of view, our data are completely in agreement with those recently reported by Foglietta et al. [11].

Of interest, among their pleiotropic functions, immunomodulatory agents thalidomide and lenalidomide, currently used as standard therapy in MM, are able to decrease Tregs number and to inhibit their proliferation and immunosuppressive function [16, 17]. Other studies, however, have instead suggested that Tregs number may be increased using these drugs [17, 18].

Finally, two groups recently found that symptomatic MM patients with long-term survival (>10 years), accounting for about 5% of all MM patients, displayed a distinct immunological profile characterized by a lower number of BM and circulating Tregs, higher Th17 cells, and proliferative cytotoxic T-cells, compared to patients with shorter survival [19, 20]. Our study does not seem to support such an observation, but the relatively short follow-up of patients should be considered.

In conclusion, our results indicate that the circulating number of Tregs does not differ among MGUS, MM, and normal subjects and does not seem to influence disease status or to provide prognostic information in MM patients as well. However, due to the contrasting results published so far, further studies are needed to better understand the possible role of Tregs in the pathogenesis and disease progression of MM, also in the light of the emerging role of novel immunotherapies, such as monoclonal antibodies, potentially active on minimal residual disease after specific treatments. Finally, the relevance of standardized methods to approach this field of investigation is also emphasized by the critical review of published data.

## Figures and Tables

**Table 1:** Main patients' characteristics at study entry.

	MM (39 patients)	MGUS (44 patients)
Age (years)		
(median; range)	69 (37–90)	65 (39–87)

Gender (number; %)		
Male	18 (46%)	25 (57%)
Female	21 (54%)	19 (43%)

International staging system (ISS) (number; %)		
ISS-1	9 (23%)	
ISS-2	19 (49%)	
ISS-3	11 (28%)	

MM/MGUS subtype (number; %)		
IgG	28 (72%)	32 (73%)
IgA	11 (28%)	8 (18%)
IgM	—	4 (9%)

Serum M-protein (g/dL)		
(median; range)	2.9 (2.1–5.3)	1.1 (0.2–1.9)

Percentage of BM plasma cells		
(median; range)	36 (23–85)	7 (1–10)

BM: bone marrow; MM: multiple myeloma; MGUS: monoclonal gammopathies of uncertain significance.

**Table 2:** Most relevant published studies investigating the frequency and prognostic significance of Tregs in MM compared with the present study.

Reference	Patients/controls evaluated	Samples tested	Method used in Treg evaluation	Tregs frequency	Functional studies	Impact on prognosis
Prabhala et al. [5]	MGUS MM Controls	Isolated PBMC	CD4^+^FoxP3^+^	Decreased	Unable to suppress anti-CD3-mediated T-cell proliferation	Not evaluated

Beyer et al. [6]	MGUS MM Controls	Isolated PBMC	CD4^+^CD25^high^FoxP3^+^ (% of CD4^+^ cells)	Increased in MM versus MGUS (trend without statistical significance)	Strong inhibitory function	Not evaluated

Feyler et al. [7]	MGUS MM Controls	Isolated PBMC and BM	CD4^+^CD25^+^FoxP3^+^	Increased in PBMC but not in BM	Not evaluated	Correlation with disease burden (paraprotein)

Gupta et al. [8]	MM	Isolated PBMC	CD4^+^CD25^+^CD127^−^FoxP3^+^ (% of CD4^+^ cells)	Reduced in untreated which increased after treatment with lenalidomide	Able to inhibit proliferation of CD4+CD25-T-cells	Increase of Tregs in responding patients to therapy; decrease correlates with ISS I + II

Muthu Raja et al. [9]	MGUS SMM MM	PB/BM whole	CD4^+^CD25^+^CD127^−^CD45RA^+/−^ (% of CD4 cells)	Increased in MM but not in SMM and MGUS	Able to inhibit the proliferation of CD4^+^ T-cells and the secretion of IFN-*γ*	Correlation with adverse clinical features (hypercalcemia, lower normal PC, and IgA subtype); no correlation with ISS; predict time to progression; MM patients with ≥5% of Tregs had inferior time to progression

Giannopoulos et al. [10]	MM Controls	Isolated PBMC	CD4^+^CD25^high^FoxP3^+^	Increased	Not evaluated	Correlation with shorter overall survival

Foglietta et al. [11]	MM MGUS Controls	Isolated freshly PB and frozen BM	CD4^+^CD25^high^FoxP3^+^	Similar	Effective suppressor function	No correlation with the pattern of BM infiltration

Present study	MM MGUS Controls	PB whole	CD4^+^CD25^high^CD127^−^ (% and absolute number)	Similar	Effective suppressor function	No correlation with laboratory and clinical variables; no correlation with outcome

BM: bone marrow; PB: peripheral blood; PBMC: peripheral blood mononuclear cells; MM: multiple myeloma; SMM: smoldering multiple myeloma; MGUS: monoclonal gammopathies of uncertain significance.
